# Supplementary material for: Exploring the Role of Peripheral Macrophages in Glioma Progression: The Metabolic Significance of Cyclooxygenase-2 (COX-2)
Source: Int J Mol Sci. 2025 Jun 27;26(13):6198. doi: 10.3390/ijms26136198 (PMC12249887; doi:10.3390/ijms26136198)
Supplement: Supplementary file 1 [file ijms-26-06198-s001.zip › ijms-3561596-supplementary.pdf]

This supplementary file has been provided by the authors to give readers additional information about the study.

Exploring the role of peripheral macrophages in glioma progression: Metabolic significance of Cyclooxygenase-2 (COX-2)

Jens Pietzsch[1,2], Magali Toussaint[3], Cornelius Kurt Donat[4], Alina Doctor[1,2], Sebastian Meister[1], Johanna Wodtke[1], Markus Laube[1], Frank Hofheinz[5], Jan Rix[6], Winnie Deuther-Conrad[3] and Cathleen Haase-Kohn[1]

Supplemental information:

Supplementary Experimental Procedures

Supplementary Figures

Supplemental Experimental Procedures

RFP control and cell cycle analysis by FACS

The RFP fluorescence control for the CRISPR/Cas9 induced COX-2 knockout was performed prior to each experiment and analyzed using the Attune NxT flow cytometer. For cell cycle analysis, ethanol-fixed cells were treated with 50 µg/mL RNase A and stained with 10 µg/mL propidium iodide or Hoechst 33342 before analysis by flow cytometry.

Determination of Prostglandin E2 (PGE_2) by UPLC-MS and ELISA

Sample processing

U87wt (2 mL per well, $5.7x10^5$ cells/mL) and U87COX-2KO (2 mL per well, $7.8x10^5$ cells/mL) cells were seeded at 48 h in six-well plates and transferred into serum-free media at 24 h before the experiment. At the beginning, cells were incubated for 2.5 h with 15 µM arachidonic acid. The supernatant was withdrawn, centrifuged (300 g, 5 min) and aliquots were taken (250 µL). For sample workup, 10 µL internal standard d4-Prostaglandin E2 (100 ng/mL) and d4-Prostaglandin D2 (100 ng/mL), 20 µL citric acid (1 M), and 2.5 µL butylhydroxytoluene (10% in ethanol) were added. The sample was extracted three times with 1 mL ethyl acetate/n-hexane (50/50), vortexed (1 min), centrifuged (300 g, 3 min) and the organic phase was collected and combined. Finally, the organic phase was concentrated to dryness in a stream on nitrogen, reconstituted in 100 µL acetonitrile/water (50/50) and analyzed by UPLC-MS/MS.

UPLC-MS

Analytical UPLC was performed on a Waters UPLC I-Class (Milford, Massachusetts, USA; binary gradient pump BSM, autosampler FTN, column manager CM, and diode array detector PDAeλ coupled to Waters Xevo TQ-S), column Aquity UPLC® BEH C18 column (waters, 100 x 2.1 mm, 1.7 µm, 130 Å), eluent: (A): 0.1 % acetic acid in ACN/MeOH 1/1, (B): 0.1 % acetic acid in H2O; flow rate 0.4 mL/min, 39.1% isocratic elution (t0 min 39.1/60.9 –t5.5 min 39.1/60.9 – t6 min 95/5 – t7 min 95/5 – t7.5 min 39.1/60.9 – t8.5 min 39.1/60.9). Multiple Reaction Monitoring (MRM) mode, which tracks specific ion transitions under optimized fragmentation conditions, was utilized for quantification of Prostglandin E2 (PGE2) and d4-Prostaglandine E2. The set-up for the Xevo TQ-S MS detector was electrospray ionization in positive mode with 150°C source temperature, 500°C desolvation

temperature, 150 L/hr cone gas, and 1000 L/hr desolvation gas (argon). Prostaglandin E2 was monitored at transitions for [M-H]- 351.19 > 271.08 (Quantifier) and 351.19 > 315.20 (Qualifier) with a cone voltage of 34 V and collision energy of 16 V.

For quantification of Prostaglandin E2, a calibration curve was generated showing a linear range of 5 – 2500 fmol on column. Prostaglandin D2, a structural very similar isomer of PGE2, was used as control for chromatographic suitability of the method and showed retention time difference of 0.5 min and baseline separation. D4-Prostaglandine E2 was used as internal standard to correct for losses during workup and analyzed using the same ionization parameters but the following transition: [M-H]- 355.22 > 275.20 (Quantifier) and 355.22 > 319.23.

ELISA

Prostaglandin E2 levels in cultured supernatants were evaluated using a commercially available enzyme-linked immunosorbent assay kit (ENZO Life Science, ADI-930-001), according to the manufacturer´s recommendations.

Clonogenic assay

A clonal growth assay was used to compare the clonogenicity after X-ray irradiation of $U87^{wt}$ vs. $U87^{COX\text{-}2KO}$. Cells were plated into 6-well plates as follows: 2,000 cells/well for sham and 2 Gy X-ray; 3,000 cells/well for 4 Gy; 5,000 cells/well for 6 Gy and 10,000 cells/ml for 10 Gy X-ray. Experiments were performed in duplicate per data point. The cells were allowed to attach for 24 hours at 37°C and immediately irradiated with X-rays using a Maxishot system (YXLON International, Hamburg, Germany; 200 kV, filtered with 0.5 mm copper). The absorbed dose was measured using a UNIDOS dosimeter (PTW, Freiburg, Germany). The dose-rate was approximately 1.1 Gy/ min at 20 mA. The cells were maintained at 37°C for 7 days to allow for the formation of colonies and then stained with 0.5% crystal violet (Sigma) in absolute methanol [57]. Colonies greater than 50 cells were counted visually under an inverted microscope (2000C, Carl Zeiss AG). Plating efficiency was calculated as ratio of the number of colonies to the number of seeded cells. Relative plating efficiency was calculated as the ratio of the plating efficiency of treated cells to the plating efficiency of sham-control × 100%. Three independent experiments, each in duplicate, were performed.

Tube formation assay

Tube formation assay for hCMEC was performed as previously described [58]. In brief, a 24-well plate was coated with Matrigel (BD Biosciences, Bedford, MA) for 30 min at 37°C. hCMEC (2×10^4 cells) were incubated in 200 µl conditioned supernatants (CS) of U87^{wt} or U87$^{COX\text{-}2KO}$, and images were captured for 24 h using a confocal time-lapse microscope (Axio Imager A1/ Carl Zeiss Jena, Germany). Immunofluorescence images after Calcein AM (Corning) staining were analyzed with the angiogenesis analysis module of FIJI to quantify tube formation of nodules, meshes, segments and junctions.

Scratch wound cell migration and invasion assay

For migration assays, 6×10^4 cells in 100 µL were seeded in a 96-well ImageLock plate (Essen BioScience; Sartorius) and allowed to adhere overnight. The scratches in 96-well format are applied using the IncuCyte® WoundMaker, a pin-based tool that applies one scratch per well, parallel in all. After the scratch, cells were washed twice, and then incubated either with 10% serum, without (0%) fetal calf serum (FCS) or conditioned medium (CM) of Mϕ. For the invasion assay, the 96-well ImageLock plate was coated with Matrigel and cells seeded and incubated overnight. On the next day, media was replaced, and cells seeded on top of the initial Natrigel layer were then covered again using by matrigel. After 30 min incubation, scratches are applied using the IncuCyte® WoundMaker. Media with (10%) and without (0%) FCS was added and the cells imaged every 12 h for 6 to 7 days. The IncuCyte® Scratch Wound Cell Migration and Invasion Software Analysis Module allows automated detection and quantification of wound properties using a 10× objective.

Brillouin shift spectroscopy

Brillouin spectroscopy is a new optical method that addresses viscoelastic properties down to subcellular resolution in a contact-free manner. Cell culture and sample preparation for the Brillouin spectroscopy was performed as previously described [59].

Immunohistochemistry of U87^{wt} and U87$^{COX\text{-}2KO}$ brain tumors

see material and method section

CD 44 immunohistochemistry of U87^{wt} and U87$^{COX\text{-}2KO}$ brain tumors

Briefly, an antigen retrieval in 10 mM heated citrate buffer in four heating cycles was performed, followed by quenching of endogenous peroxidase and endogenous biotin in 3% (v/v) hydrogen peroxide for 10 min and by using Biotin-Blocking System from Dako according to manufacturer's instructions, respectively. Tissue sections were incubated in 5% BSA and 1% Tween in TBS-T for 1 h to block unspecific binding and, hereinafter, incubated with primary antibody CD44 (Abcam, #157107) or isotype control 1/2000 at 4 °C over night. An incubation with a biotinylated secondary antibody against rabbit for 1 h followed. For visualization, sections were incubated with ExtrAvidin peroxidase (Sigma Aldrich, 1:50) for 30 min and AEC substrate kit (BD Biosciences, 1:50) for 2–5 min. Tissue sections were counterstained with Mayer's hematoxylin, embedded in aqueous solution and imaged using AxioImager.A1 microscope with AxioVision software (version 4.8, Carl Zeiss). Immunohistochemical staining was quantified using ImageJ/FIJI (version 1.54i; National Institutes of Health). Therefore, the images were converted into 8-bit RGB images. Then a color threshold was defined: RGB values were set for cell nuclei (see table) and immunohistochemical positively stained areas (see table). The thresholded area was converted into a black and white binary image. Therefrom, the area (px^2) was determined using the analyze particle tool. The ratio of the originally red signal area to the area of the cell nuclei (originally blue) was analyzed.

Sample	Color of interest	Red	Green	Blue
Tumor	Positively stained areas (all ranges included)	92-255	0-228	15-155
	Nuclei (red range excluded, Green and Blue included)	187-255	27-255	7-255
spheroids	Positively stained areas (all ranges included)	197-255	108-255	136-255
	Nuclei (red range excluded, Green and Blue included)	83-255	0-164	17-130

Supplemental Figures

COX-2 knockout detection via RFP using FACS

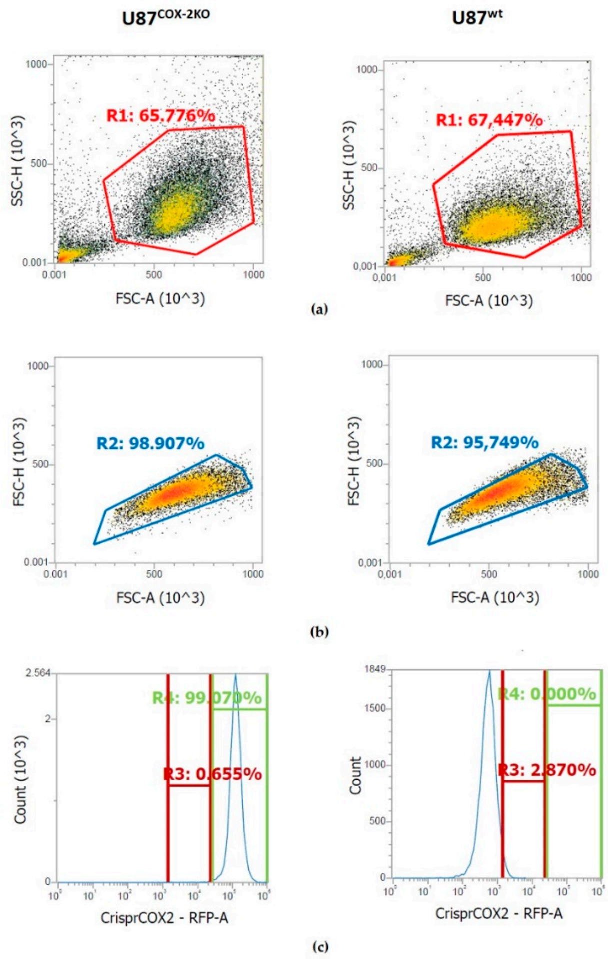

Supplementary Figure S1: Representative FACS images of U87$^{COX\text{-}2KO}$ (left) and U87^{wt} (right) cells. Flow cytometry (Attune Nxt Flow Cytometer) was used to quantify the expression levels of RFP, as well as the percentage of COX-2 knockout cells. For the identification of RFP positive cells, (**a**) the scatter characteristics (forward scatter, FSC; and side scatter, SSC) were used for gating of all events. (**b**) Singlets gating was performed using FSC-H (high) and FSC-A (area) scatter and appear on a diagonal in FSC-H/FSC-A dot plot. (**c**) RFP sorting was performed using a laser with an excitation wavelength of 561nm.

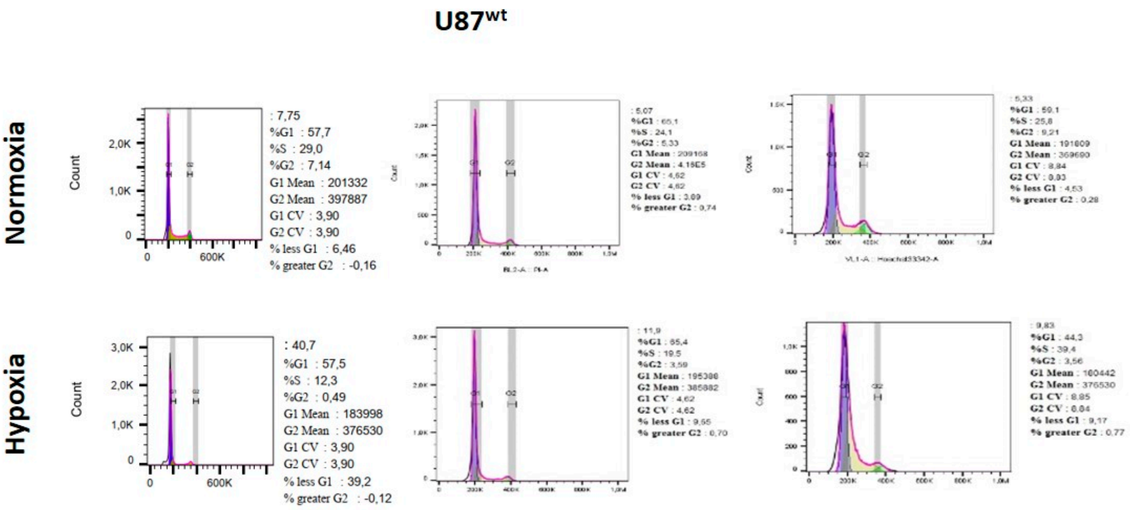

(d)

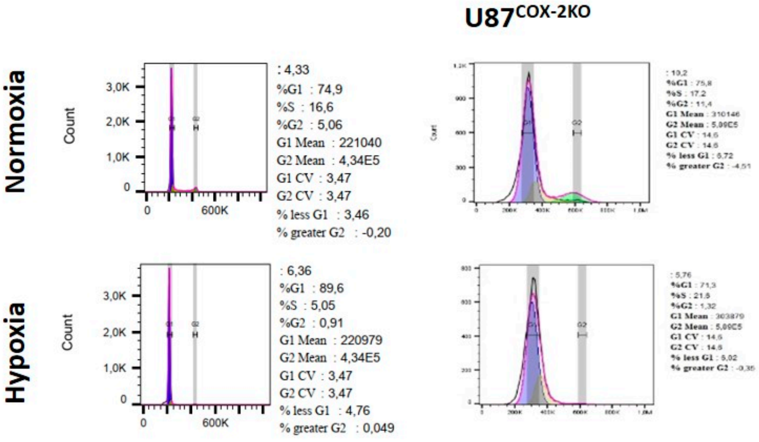

(e)

Supplementary Figure S1 continued: Cell cycle analysis of U87^{wt} (d) and U87$^{COX\text{-}2KO}$ (e) cells under normoxic and hypoxic (24h) conditions, and analyzed by FlowJo 7.6.1 software (FlowJo, LLC, Ashland, OR, USA).

Prostaglandin E2 (PGE_2) release is unaltered by COX-2 knockout

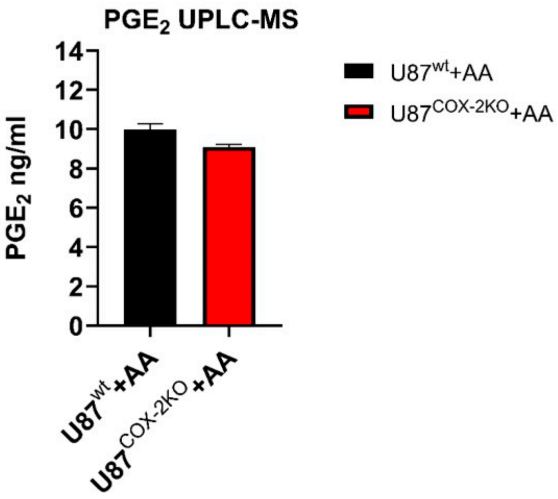

(a)

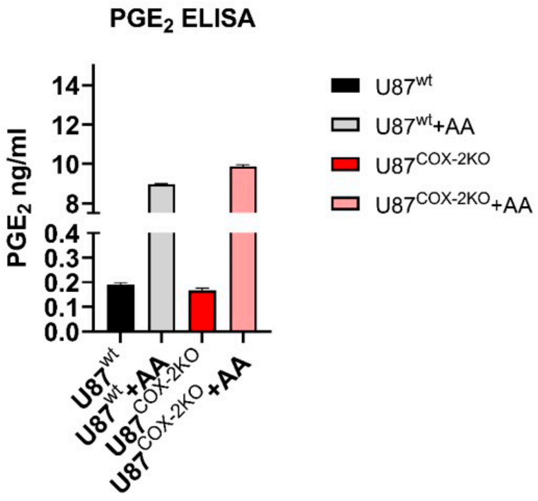

(b)

Supplementary Figure S2: (**a**) Analytical UPLC-MS of supernatants produced by U87^{wt} and U87$^{COX\text{-}2KO}$ cells after incubation with 15 μM Arachidonic acid (abbreviated with "AA") for 2.5 hours. (**b**) PGE_2 ELISA from culture supernatant. Here, cells were also incubated with 15 μM Arachidonic acid for 2.5 hours. Data represent the mean ± SEM with n = 4.

COX-2 knockout cells show reduced clonogenic survival

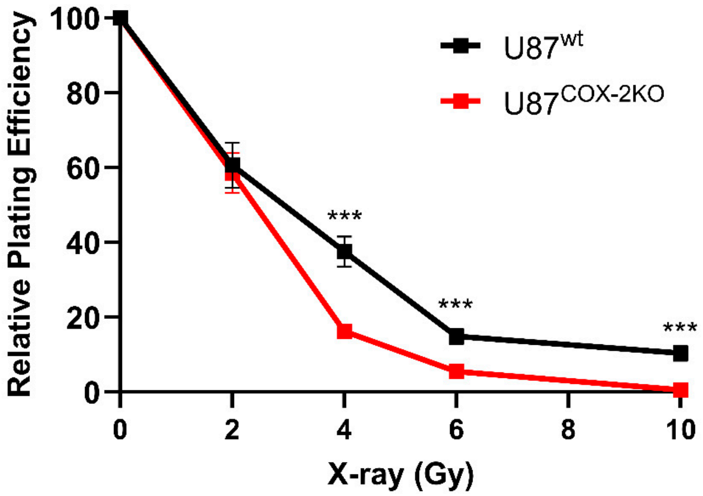

Supplementary Figure S3: Relative plating efficiency after X-ray. Here, plating efficiency of untreated U87^{wt} and U87$^{COX\text{-}2KO}$ cells were set to 100% and the relative plating efficiency after irradiation with 2, 4, 6, and 10 Gy was determined. Data are shown as mean ± SEM of three independent experiments (statistically significant with ****P<0.001*).

COX-2 knockout cells restrain angiogenesis network formation of hCMEC

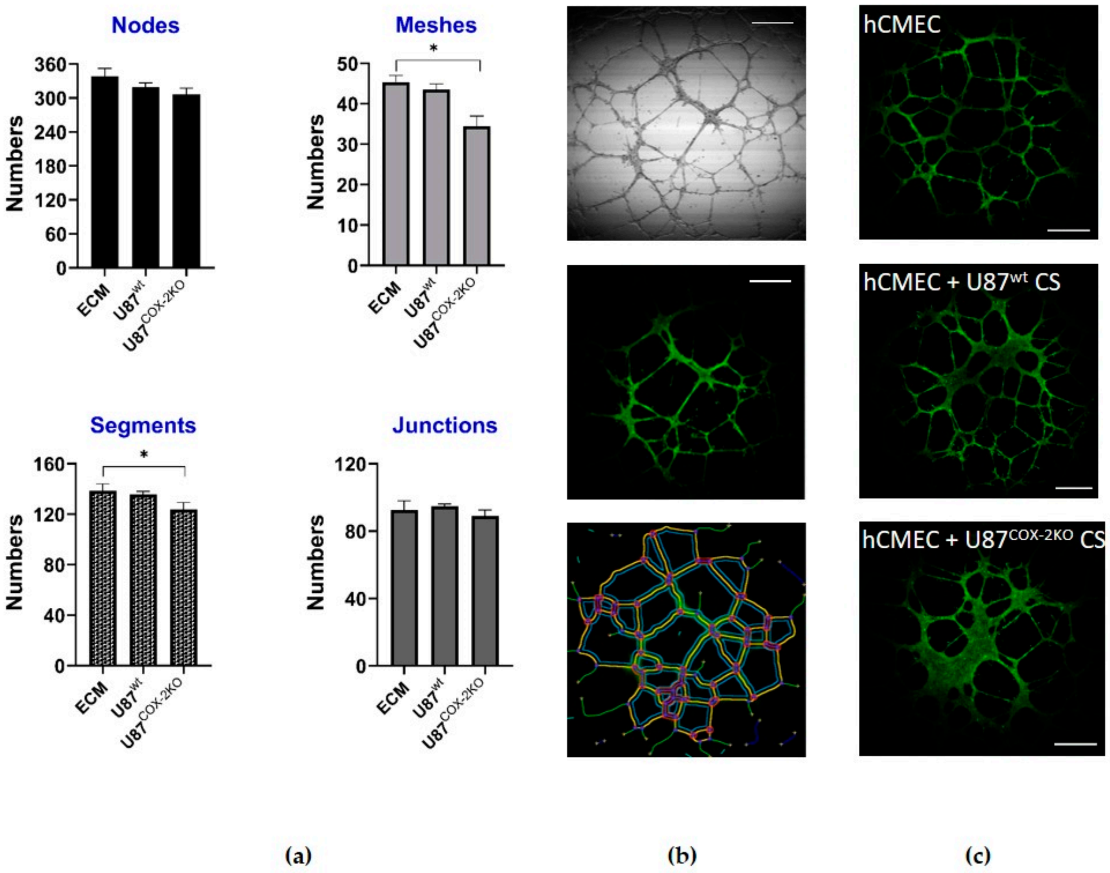

Supplementary Figure S4: (**a**) Quantitative analysis of nodes, meshes, segments and junctions formed by hCMEC, compared to hCMEC treated with culture supernatants (CS) of U87^{wt} and U87$^{COX\text{-}2KO}$ cells. Analyzed with the angiogenesis analyzer module in *FIJI*. Data represents mean ± SEM, n = 3. * $P< 0.05$ as determined by Student's t-test. (**b**) Representative images of hCMEC vascular network analysis in culture after 24 h with Calcein-staining (green). Measurement are based on the recognition of vector objects, i.e. segments (yellow), junctions (magenta, framed circular dots; group of fusing nodes), meshes (blue) and nodes (red, circular dots). Scale bar: 500 µm. (**c**) Confocal immunofluorescence of representative angiogenesis images after calcein-staining of hCMEC, and hCMEC treated with culture supernatants (CS) of U87^{wt} and U87$^{COX\text{-}2KO}$ cells. Scale bar: 500 µm.

COX-2 knockout increases cell invasion and motility

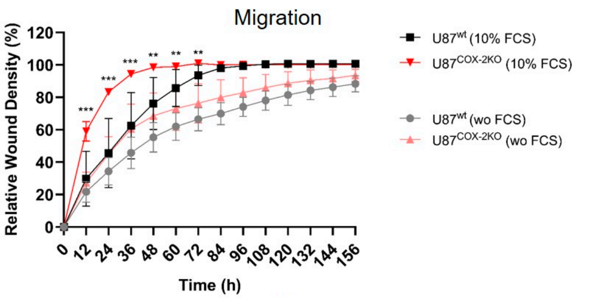

(a)

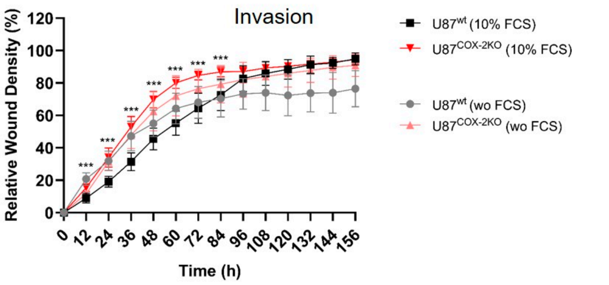

(b)

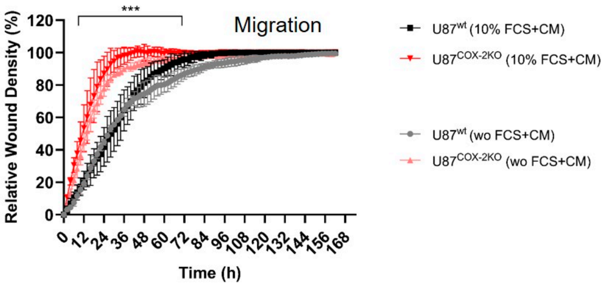

(c)

Supplemental Figure S5: (**a**) The migration potential and (**b**) the invasion properties through Matrigel were analyzed in a scratch wound healing assay over 7 days. (**c**) The migration behavior was further analyzed after incubation with conditioned medium (CM) of Mϕ. Cells were imaged under phase contrast (10x) every 12 hours around wounds using the IncuCyte® Live-Cell Monitoring System. Data are shown as mean ± SEM of three independent experiments (statistically significant with **P<0.01* and ***P<0.001*).

Increased viscoelastic properties in U87^{wt} spheroids

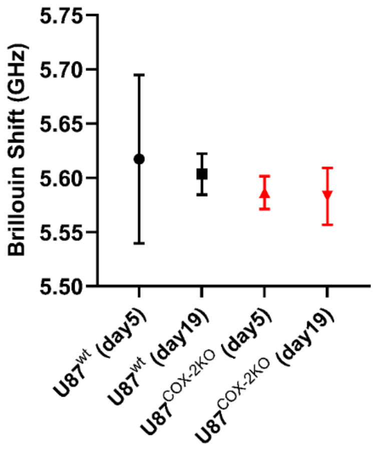

Supplementary Figure S6: Brillouin shift frequencies of U87^{wt} and U87$^{COX\text{-}2KO}$ spheroids after 5 and 19 days in culture (n=10).

Effect of staining intensity, total cell density in tumors and quantitative histology of the stroma (150 μm from tumor border)

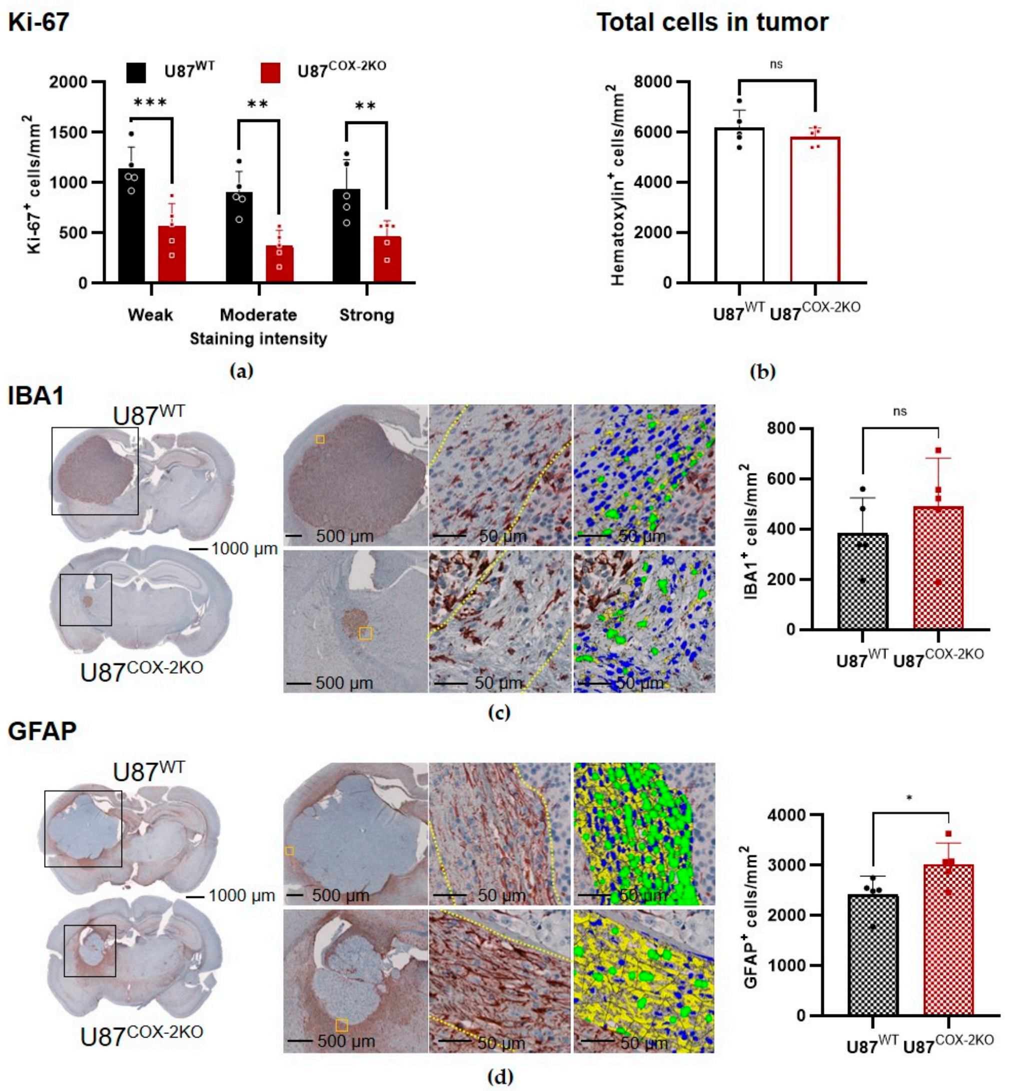

Supplementary Figure S7: (**a**) Number of Ki-67-positive cells per mm² classified either as weak, moderate and strong across U87^{wt} and U87$^{COX-2KO}$ orthotopic tumors. Two-way ANOVA indicates a main effect of genotype, but not staining intensity.

(**b**) Total number of hematoxylin-positive cells per mm² does not differ between U87^{wt} and U87$^{COX-2-KO}$ cells, indicating that significant changes in Ki-67- and GFAP-positive cells are not caused by differences in density. Representative images and quantitative histology of the tumor border zone of U87^{wt} and U87$^{COX-2KO}$

orthotopically implanted cells, stained with IBA1 (**c**) and GFAP (**d**). A black rectangle indicates the magnified area shown in the middle-left panel. A further magnification and its location is provided in the middle-right panel, along with the ROI spanning 150 μm from the tumor border (yellow dotted line). The right panel shows the segmentation results as color-coded overlay, with hematoxylin-positive (blue) and IBA1/GFAP-positive cells (green). Processes are labelled yellow (quantified, but not analyzed).

Graphs show the mean±SD of stain-positive cells per animal (from 6-12 sections) of U87^{wt} and U87$^{COX2\text{-}KO}$ (n=5 each). Scale bars are presented in each photomicrograph.

* P<0.05, significant differences of U87^{wt} and U87$^{COX2\text{-}KO}$, tested with an unpaired two-tailed t-test.

Quantitative histology – isotype control

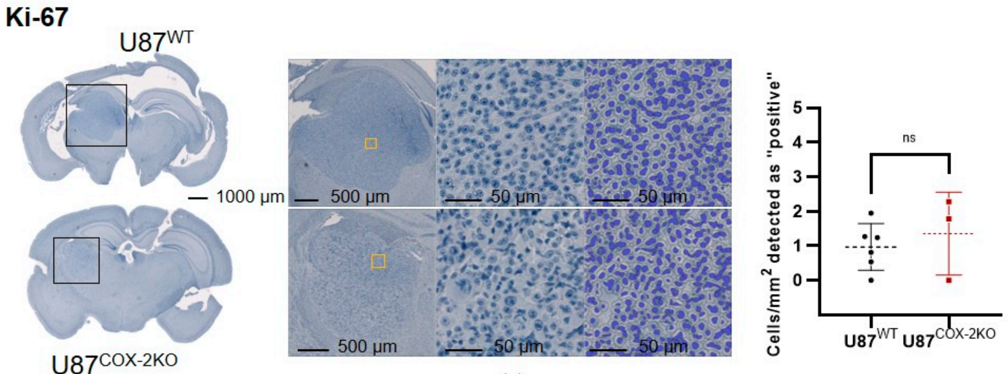

(a)

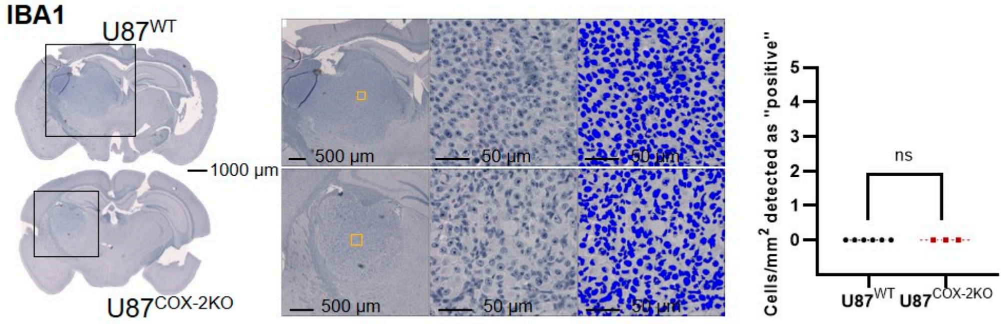

(b)

Supplementary Figure S8: Isotype controls were performed on adjacent FFPE tissue sections used for quantitative histology to confirm accuracy of staining and quantitative histology.

Representative images and quantitative histology of the isotype controls for U87^{wt} and U87$^{COX\text{-}2KO}$ tumors, stained with Ki-67 (**a**) and IBA1 (**b**). A black rectangle indicates the magnified area shown in the middle-left panel. A further magnification and its location is provided in the middle-right panel, with segmentation results in the left panel.

(**a**) CytoNuclear segmentation performed on isotype control staining for Ki-67. Graph shows the number of false positive cells per mm^2 for U87^{wt} and U87$^{COX\text{-}2KO}$.

(**b**) Microglia module segmentation performed on isotype control staining for IBA1. Graph shows the number of false positive cells per mm^2 for U87^{wt} and U87$^{COX\text{-}2KO}$.

Graphs show the mean±SD of false positives per animal (from 6-12 sections) of U87^{wt} and U87$^{COX2\text{-}KO}$ (n=5 each). Scale bars are presented in each photomicrograph.

Quantitative histology – control using % area

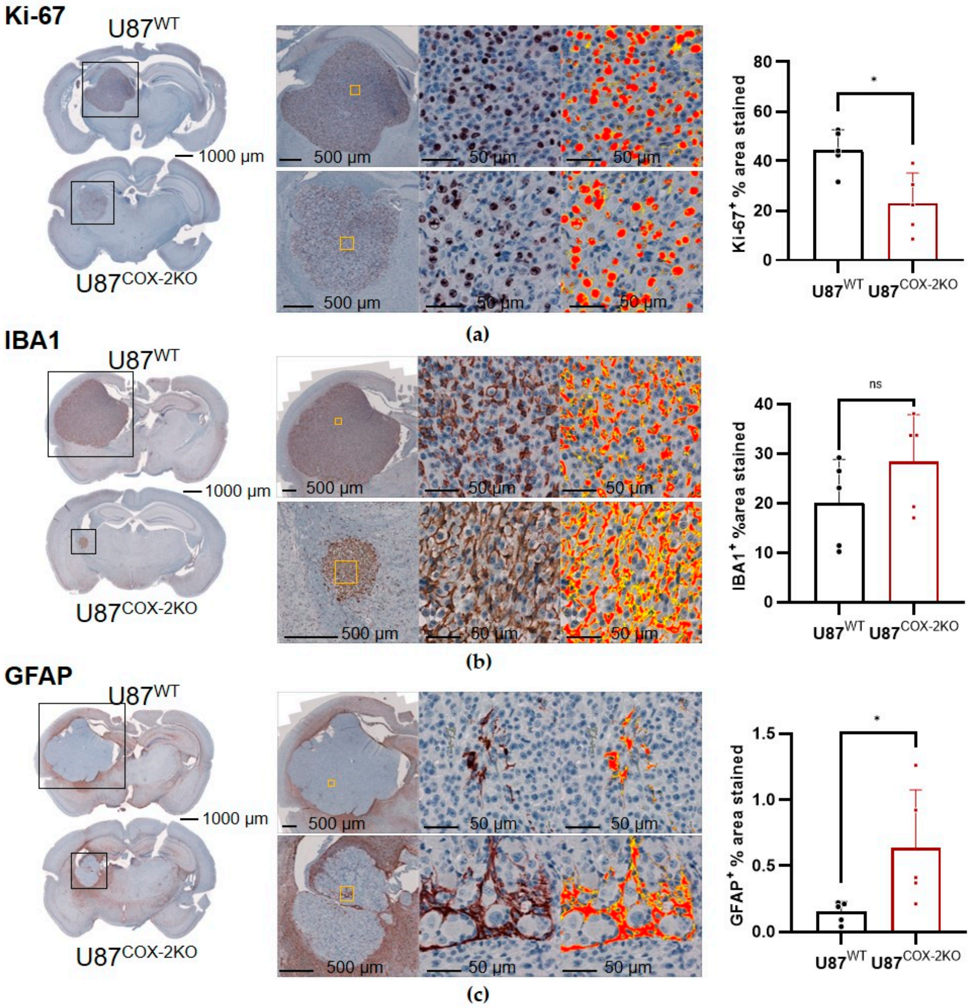

Supplementary Figure S9: To further confirm accuracy of the segmentation-based quantitative histology, we also performed measurements using percent area covered by stain, with graph showing the quantification. Representative images and quantification using % area stained for U87^{wt} and U87$^{COX\text{-}2KO}$ tumors, with Ki-67 (**a**), IBA1 (**b**) and GFAP (**c**). A black rectangle indicates the magnified area shown in the middle-left panel. A further magnification and its location is provided in the middle-right panel, with % area stained results (red=strong; orange= moderate, yellow=weak) in the left panel.

(**a**) Ki-67, percent area covered by stain.

(**b**) IBA1, percent area covered by stain.

(**c**)GFAP, percent area covered by stain.

Graphs show the mean±SD of percent area covered by stain per animal (from 6-12 sections) of $U87^{wt}$ and $U87^{COX2-KO}$ (n=5 each). Scale bars are presented in each photomicrograph.

* P<0.05, significant differences of $U87^{wt}$ and $U87^{COX2-KO}$, tested with an unpaired two-tailed t-test.

COX-2 knockout decreases CD44 staining

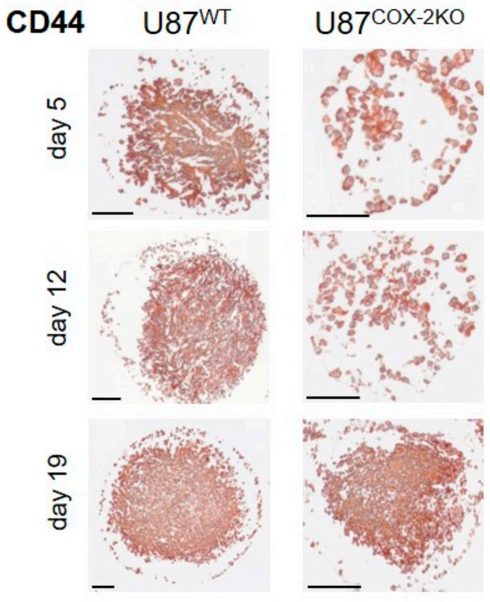

(a)

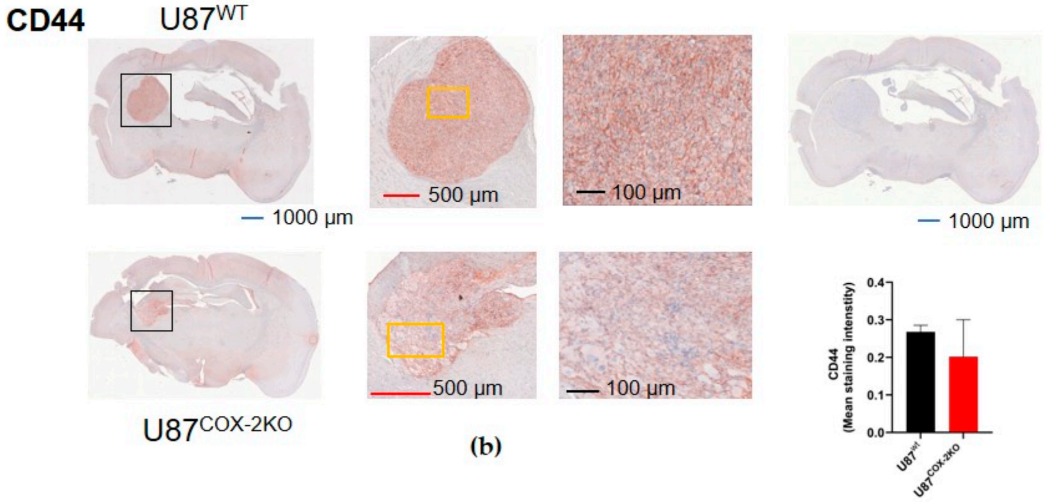

(b)

Supplementary Figure S10: (**a**) Representative images of spheroids after day 5, 12, and 19 with staining for CD44. Scale bar: 100 µm. (**b**) Representative images of tissue sections stained for CD44 from animals orthotopically implanted with U87^{wt} and U87$^{COX\text{-}2KO}$ orthotopically implanted cells and quantitative analysis (n=2), stained with CD44. A black rectangle indicates the magnified area shown in the middle-left panel. A further magnification

and its location is provided in the middle-right panel. The right panel shows the histology of the isotype controls for U87^{wt} and U87$^{COX-2KO}$ tumors. Scale bars are color-coded with light blue: 1000 μm (whole brain photomicrographs); red: 500 μm; solid black: 100 μm.
